# Competitive ability is a fast-evolving trait between house mouse populations (*Mus musculus domesticus*)

**DOI:** 10.1186/s12983-022-00476-7

**Published:** 2022-12-08

**Authors:** Miriam Linnenbrink

**Affiliations:** Max-Planck Institute for Evolutionary Biology, August-Thienemann Str. 2, 24306 Plön, Germany

**Keywords:** *M. m. domesticus*, House mouse, Populations, Territoriality, Competitive ability, Fitness, Semi-natural enclosure

## Abstract

**Background:**

House mice are commensal animals with a nearly global distribution, structured into well differentiated local populations. Besides genetic differences between the populations, they have also diverged behaviorally over time, whereby it remains open how fast general behavioral characteristics can change. Here we study the competitive potential of two very recently separated populations of the Western house mouse (*Mus musculus domesticus*) by using two different approaches—one under controlled cage conditions, the other under more natural conditions in enclosures mimicking a secondary encounter condition.

**Results:**

We observe a clear bias in the competitive ability towards one of the populations for both tests. The measured behavioral bias is also reflected in the number of hybrid offspring produced in the enclosures.

**Conclusion:**

Our data suggest that key behavioral characteristics with a direct influence on relative fitness can quickly change during the evolution of populations. It seems possible that the colonization situation in Western Europe, with a rapid spread of the mice after their arrival, would have favored more competitive populations at the expansion front. The study shows the possible impact of behavioral changes on the evolution of populations.

**Supplementary Information:**

The online version contains supplementary material available at 10.1186/s12983-022-00476-7.

## Background

Competitive interactions between individuals occur usually in the context of resource allocation [1–3], both within, as well as between species. These resources can be general habitat characteristics, food and nesting sites and, in intraspecific competitions, the competition for potential mating partners. The competitive potential of individuals eventually influences survival and reproductive success [4–10]. Apart of the effects on individuals, it can also have a general effect on population growth and range expansion [11–13]. To have an overall effect on the population, it is important to show that it is not due to behavioral plasticity of single individuals but has a general genetic basis in the respective population [14]. For the individual, the ability to successfully compete against others serves as basis to defend resources if they are restricted to a certain area (i.e. territory). The associated behavior is called “territoriality” [2, 15, 16], which has a long history of investigation in various taxa and species [17].

Mice are commensal animals which spread across the world in the wake of human colonization [18, 19]. They are highly social and live together in big family groups with established hierarchies [8, 20–22]. Behavioral studies of wild house mice have focused on mate choice, vocalization behavior or territoriality [21, 23–27]. Also competitive ability has been studied within and between different subspecies of mice [28–31], mostly performed in controlled cage experiments, though. As mice are known to live in a demic structure [8, 21, 22, 32] which come into contact when individuals disperse and demes grow, competitive behavior between individuals is likely an important factor for the evolutionary change in mouse populations.

Here we study competitive ability as an important behavioral trait in the evolutionary divergence of populations. We use wild–derived individuals from two allopatric populations of the Western house mouse, one caught in the Massif Central (France) and the other caught in the area of Cologne/Bonn (Germany). The Western house mouse (*Mus musculus domesticus*) has colonized Europe about 3,000 years ago and the populations are likely to have been separate since. They show clear genomic differentiation [32–34], differences in gene expression [35, 36], ultrasonic vocalization and mate choice [25, 26]. To investigate the competitive potential of animals from either population, we performed controlled cage experiments with one-by-one encounters of male mice. As a second test, we used enclosures in which mice could set up their own territory, before connections to the other population were opened. The questions we ask are: a) Are individuals of both populations equal in their competitive ability and b) is a higher competitive ability related to higher resource allocation in terms of access to females of the respective other population.

## Results

### Competitive potential in the encounter experiments

15 male-male dyads, consisting of one male of MC and one of CB origin, were tested in one by one encounter experiments. CB animals were on average significantly heavier than MC animals, 29.5 versus 26.8 g (Wilcoxon test: W = 64, *p* = 0.045), but the dyads were arranged such that weight differences were not biased towards CB individuals (see Methods) and making weight differences between the pairs non-significant (Wilcoxon test: V = 39, *p* = 0.252) (Table 1).

**Table 1:** Number of decided conflicts per individual of each dyad

Dyad	Population	Weight [g]	Age [days]	# of decided conflicts	Population	Weight [g]	Age [days]	# of decided conflicts	total # of decided conflicts	Binomial test *p*-value
1	CB	33.64	288	28	MC	19.89	313	2	30	< 0.001
2	CB	34.02	306	0	MC	20.57	301	0	0	–
3	CB	34.75	318	0	MC	21.64	313	24	24	< 0.001
4	CB	36.56	326	27	MC	21.84	401	0	27	< 0.001
5	CB	36.77	315	39	MC	24.08	331	0	39	< 0.001
6	CB	23.91	326	0	MC	27.94	309	28	28	< 0.001
7	CB	25.06	293	12	MC	28.4	331	1	13	0.003
8	CB	25.38	326	25	MC	31.17	309	1	26	< 0.001
9	CB	26.6	319	0	MC	33.36	309	24	24	< 0.001
10	CB	27	344	22	MC	35.83	383	2	24	< 0.001
11	CB	24.53	319	0	MC	24.27	382	0	0	–
12	CB	28.21	293	8	MC	24.73	383	0	8	< 0.001
13	CB	28.62	316	6	MC	25.62	331	0	6	< 0.001
14	CB	28.7	288	11	MC	26.65	343	0	11	< 0.001
15	CB	28.71	290	11	MC	26.89	401	0	11	< 0.001
Total	CB	29.50	311.13	189	MC	26.19	342.67	82	271	

We found no significant differences in conflict numbers between the test of the first and the second day for the dyads (Wilcoxon test: V = 39.5, *p* = 0.255), hence we pooled the numbers across days. Over all pairs and days we observed 638 conflict situations, ranging from 0 to 76 conflicts per pair. Of these, 271 conflicts were classified as “decided” (42.4%), which are the basis for determination of a competitive bias between the individuals of the two populations.

In 13 out of 15 dyads decided conflicts occurred, which means that in 86.7% of tested dyads showed a clear hierarchy between both individuals (Binomial test, *p* = 0.005). In each of the 13 dyads one individual could be determined as “winner” (*i.e.* dominant) as the number of decided conflicts differed significantly between the two individuals of each dyad (for results of binomial tests see Table 1). Overall, in 10 of 13 dyads the CB individuals were predominant over MC individuals (Binomial test: *p* = 0.048).

**Fig. 1:**
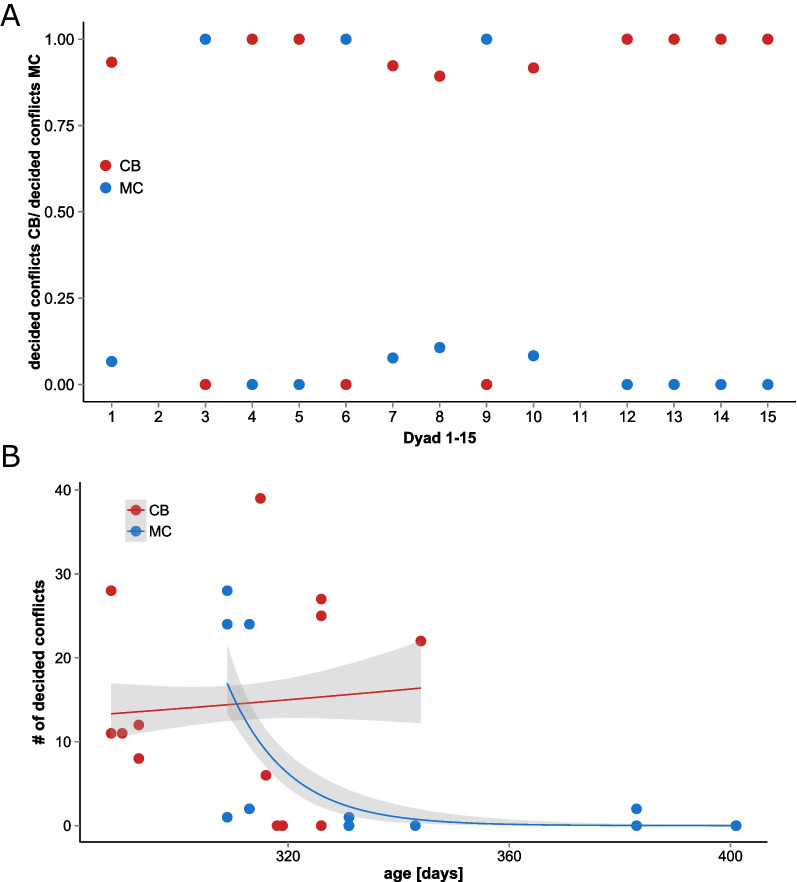
**A** Competitive potential between male individuals of the two populations MC and CB for all observed dyads. Note that dyads 2 and 11 did not show any decided conflict. **B** Interactive effect of population background and age of mice on the number of decided conflicts

Visualization of the number of decided conflicts per dyad (Fig. 1) shows that, if a competitive bias is present between both dyadic partners, it is explicit. No intermediate forms of competitive bias occurred in the observed encounters.

By using a zero inflated negative binomial poisson regression model we tested if population background (either MC or CB) and/or the age and/or the body weight of the focal mice had a significant influence specifically on the outcome (number of conflicts won) of the encounter tests. CB individuals won significantly more conflicts than MC individuals (z = 2.651, *p* = 0.008). Figure 1A shows that, if a competitive bias is present between both dyadic partners, it is explicit. No intermediate forms of competitive bias occurred in the observed encounters apart of the two individuals where no decided conflict was observed. Further, we detected an interactive effect of age and population background to have a significant effect on the potential to win conflicts (z = − 2.433, *p* = 0.015, Fig. 1B), i.e. mice with MC population background lose their competitive potential by aging. In mice from the CB population, this effect was not observed. Body weight did not influence the dyadic encounters, as expected due to arranging the weights.

**Fig. 2:**
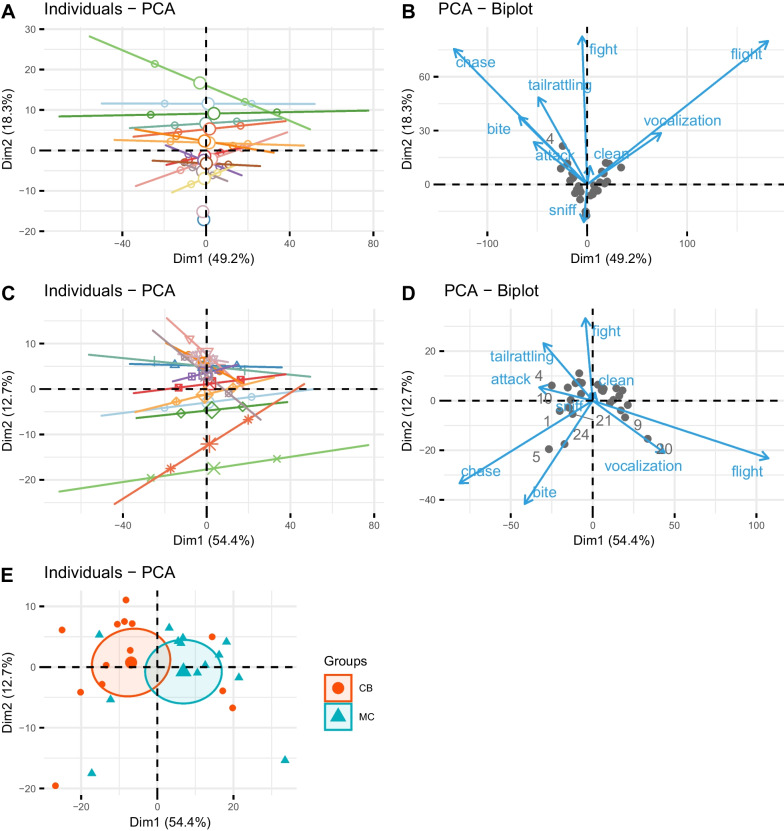
Two first components of the PCA on the behaviors which contributed > 5% to overall behaviors. **A** individual PCA results for all 30 individuals, where individuals belonging to one dyad are connected via a solid line, small open circles represent individuals’ scores, large open circles represent the average of both dyad partners, **B** PCA—biplot showing the direction and strength of behaviors for the two first components, where more offensive behaviors are directing to the left side on the first component and defensive behaviors directing to the right (*n* = 30).Thus, taking A and B together, the more competitive successful individuals can be inferred to be located on the left side of the individuals average and less successful individuals on the right. **C** and **D** are analogous to A and B, for only those 26 individuals (13 dyads) showing distinctive competitiveness determined by “winner”/”loser” assignment based on the number of decided conflicts. **E** individual PCA grouped by population background, again, on the left side of the X-Axis, the more competitive individuals, belonging mainly to the CB population versus the mostly less competitive individuals on the right side of the average

Next, we used Principal Component Analysis, where we included more observed behaviors than included in the “decided”/”undecided” analysis. All behaviors which accounted for > 5% of all behaviors observed were included, as e.g. fight, flight, chase, tail rattling, attack, cleaning, sniffing, vocalization and biting. PCA for all 15 dyads showed a clear distinction of both partners for 13 of 15 dyads on the first axes, which accounted for 49.2% (Fig. 2A). The two dyad partners where no decided conflicts occurred also show no separation on this first PCA component (the two lowest points in light blue and pink in Fig. 2A). Here we confirmed the neutrality of those two pairs also by extending the analyzed behaviors by tail rattling, cleaning, sniffing, vocalization and biting. In most cases, dyad pairs use certain behaviors more often than others—which contributes to a separation on Axis 1 for more offensive and defensive behaviors (Fig. 2B). Sniffing separates on the second component and is mostly used by the two neutral dyads.

A PCA for non-neutral dyads (Fig. 2C) revealed a clear differentiation of dyad partners on axis one (54.4%), which seems to be an axis describing competitive behavior as individuals with positive coefficients tend to flee more often and vocalize more, individuals with negative coefficients have a higher frequency of tail rattling, attack more often, box and chase more often (Fig. 2D). Grouping individuals by population background shows MC individuals to be more submissive and CB individuals more aggressive—however, clusters do overlap (Fig. 2E). This result is in clear concordance with the simple “winner”/ “loser” assignment based on the number of decided conflicts. However, ANOSIM analysis underpins this observation as a significant influence of population background on behavioral differences between both populations (R = 0.17, *p* = 0.018).

### Influence of competitive differences on space use

**Fig. 3:**
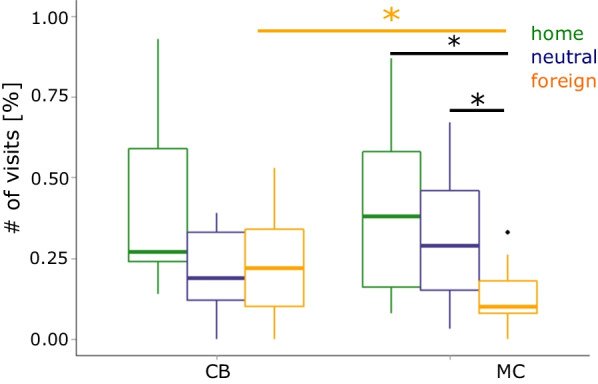
Cage use in the encounter experiments of CB versus MC individuals (*n* = 15). Boxplots show show the medians (horizontal lines), as well as the 25% and 75% quartiles, whiskers represent extreme values and open circles indicate outliers

Counting the visits of the different setup compartments of each individual across all experiments reveals that the three main cages (home cage, neutral cage and foreign cage) had most visits (mean 43.4%, 24.1% and 27.6%, respectively), less in the plexiglas tubes (9.2%) and the water bath or escape cage (6%). To determine the influence of the competitive behavior on spatial use in the experimental setup, we took only those 13 dyads into account, which showed significant differences in their competitive potential. The analysis on space uses focuses on the use of the three main cages (home, neutral and foreign) (Fig. 3). A Friedman Rank Sum test was used to test whether the three main cages were visited equally often, this was not the case overall (Friedman test: N = 30, X^2^ = 16.2, df = 2, *p* < 0.001).

One can see an overall population effect for cage use (Fig. 3) (for this calculation we include also the two dyads, where no hierarchy between both individuals was established). CB animals use all three main cages equally frequent (Friedman test: N = 15, X^2^ = 6.93, df = 2, *p* = 0.031) compared to MC animals, which show unequal use of the three main cages (Friedman test: N = 15, X^2^ = 9.39, df = 2, *p* = 0.009). MC individuals use the foreign cage significantly less than the home and neutral cage (Wilcoxon test: home vs. foreign: N = 15, *p* = 0.021 and neutral vs. foreign: N = 15, *p* = 0.001). The use of the foreign cage differed significantly between mice of the two populations (Wilcoxon test: N = 15, V = 6, *p* = 0.003 (after Bonferroni correction)).

### Population development in the enclosure experiment

The enclosure experiments sheds light on the difference of competitive ability between mice from two house mouse populations under more natural conditions, *i.e.* after having established their own territory. We aimed for almost equal frequencies of adult MC and CB individuals at the start of the experiment (see Table 2), however, both rooms showed a little skew towards the total number of individuals of the MC population, as several young individuals were still living with their mothers and could not be removed at the start of the experiment.

**Table 2:** Population development of the enclosure experiments in room #113 and room #114

			Room #113		Room #114	
Phase	Population	Age	# of individuals	Genotype frequency	# of individuals	Genotype frequency
START	MC	Adult	27 (14/13)	56.3%	31 (14/17)	55.8%
		Young	22 (0/0/22)		12 (7/5)	
	CB	Adult	23 (10/13)	43.7%	26 (11/15)	44.2%
		Young	15 (1/10/4)		18 (13/5)	
HEALTH CHECK	MC	Adult	34 (18/16)	37%	42 (16/26)	37.2%
		Young				
	CB	Adult	35 (16/19)	38.0%	45 (20/25)	40%
		Young				
	N/A	Adult	–	25%	–	23.0%
		Young	23 (8/12/3)		26 (11/15)	
END	MC	Adult	30 (16/14)	51.7%	40 (15/25)	24.0%
		Young	45 (20/25)		8 (3/4/1)	
	CB	Adult	34 (15/19)	33.8%	42 (20/22)	47.0%
		Young	15 (8/7)		52 (26/26)	
	MIX	Adult	–	13.1%	–	23.0%
		Young	19 (11/8)		46 (13/22/11)	
	N/A	Adult	–	1.4%	–	6.0%
		Young	2 (1/1)		12 (4/5/3)	

The number of individuals is given for three time points (1) at the beginning (2) during the health check and (3) at the end of the experiment. Counts are given for adult (with RFID tag) and young (only ear clip, without transponder) individuals separated by population background (i.e. pure MC, pure CB or mixed population background)

Numbers for females and males are given in brackets and denoted as (males/females/unknown sex). Further, genotype frequencies per population per room and time point were calculated

During the health check after 4 weeks, the first offspring was tagged and ear clipped.

**Fig. 4:**
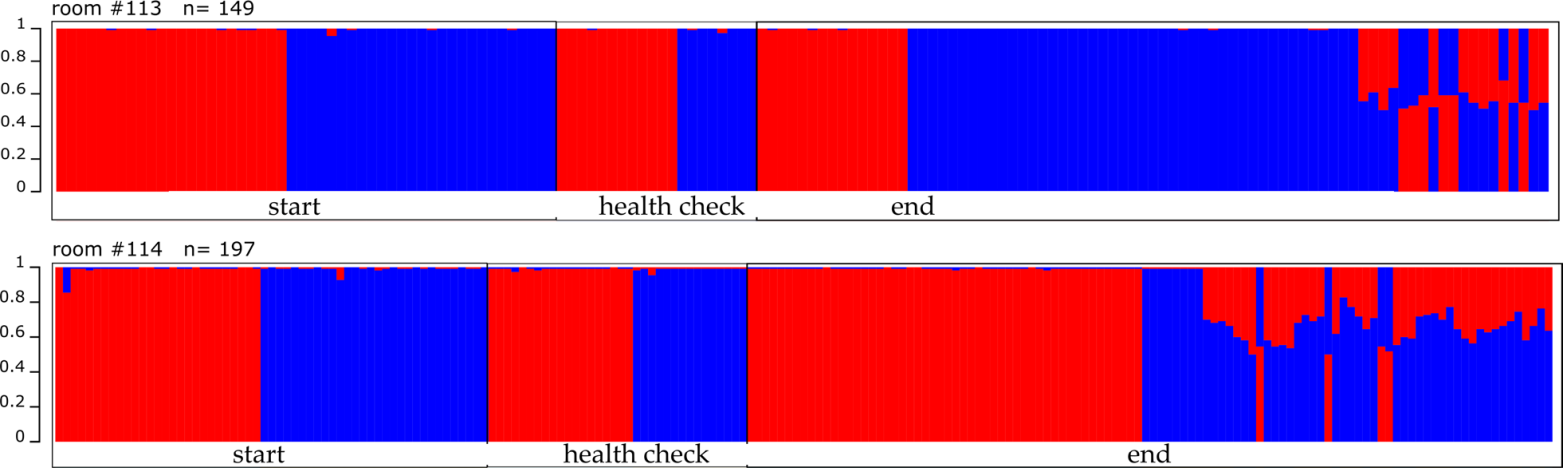
Population origin of the animals in the enclosure experiment. Structure plot for all sampled individuals of room #113 (upper panel) and room # 114 (lower panel), each bar represents a single individual. Red and blue indicate the population origin; MC = blue, CB = red. Founder individuals and individuals sampled during the health check were either of pure MC or CB origin, respectively. Offspring individuals with mixed population background only occurred at the end of the experiment

By using the program STRUCTURE [37, 38] we assessed the population origin of all individuals. We revealed that the individuals tagged during the health check were all of pure population origin (pure MC or pure CB, Fig. 4) and were sired during the phase before opening the arena connections, or were already born at the start of the experiment but too young to receive an RFID transponder. This is also the reason, why we did not differentiate between adult founder individuals and offspring tagged during the health check in further behavioral analysis. However, during the health check, also pups have been found, which were not ear clipped (see Table 2), i.e. it is open if they still result from within-population breeding or from early hybridization between the two populations.

Individuals which could not be sampled and tagged during the health check are included in the final offspring numbers and population origin was determined by STRUCTURE analysis [37, 38]. In the final offspring individuals of pure MC and CB origin were found, but also individuals with mixed population background (room #113: *n* = 19; room #114: *n* = 46, see Table 2 and Fig. 4). We focused the further analysis on these mixed individuals, since we aimed to investigate if there is a higher competitive potential of one of the populations, and if so, if there is a different use of resources (e.g. space use and access to females of the respective other population).

### Paternities in the arenas

We inferred maternal and paternal population origin of mixed offspring by using the program CERVUS [39]. We used the option to identify the two most likely parents. If the first and second most likely father/mother came from the same population (or even was the same individual), we accepted the population background of that individual as being the maternal/paternal background of the analysed offpring.

For 44 of 65 mixed background offspring individuals, paternal population origin can be attributed to CB males and 8 individuals to MC males (see Table 3). For 13 individuals, paternal/maternal population origins could not unequivocally be determined. A test for equal proportions of the total number of offspring sired by males of the one or the other population revealed a trend difference for room #113 (*p* = 0.077), in room #114 the proportions are significantly different (*p* < 0.001), however, overall CB males sired more mixed offspring than MC males (*p* < 0.001).

**Table 3:** Number of offspring sired by CB or MC males

Paternal background	Room #113	Room #114
CB	11	33
MC	5	3
Undetermined	3	10
SUM	19	46

### Curiosity and dispersal of mice in the enclosures

We estimated curiosity and dispersal of mice based on the antenna data using two measures. First, the time individuals needed to discover the pipes to the other arena (touch latency) and the duration after the registration at one antenna for the first time and the registration for the first time at one antenna in the foreign arena (cross latency). Both measures are positively correlated for all mice harboring a transponder (Pearson correlation: r = 0.86, *p* < 0.001). As weight could influence competitive outcomes, we first tested for differences in weight of MC (*n* = 70) and CB (*n* = 75) individuals and found no significant difference (Wilcoxon test: W = 2498.5, *p*-value = 0.6181). In addition, we performed a PERMANOVA with population, sex and weight as explanatory variables and touch/cross latency, as well as number of sessions and the time spent in the foreign arena. Population background is the only factor significantly influencing competitive and exploratory behavior of mice (population: F = 117.6, *p* = 0.007; sex: F = 3.1, *p* = 0.46; weight: F = 17.5, *p* = 0.16).

**Fig. 5:**
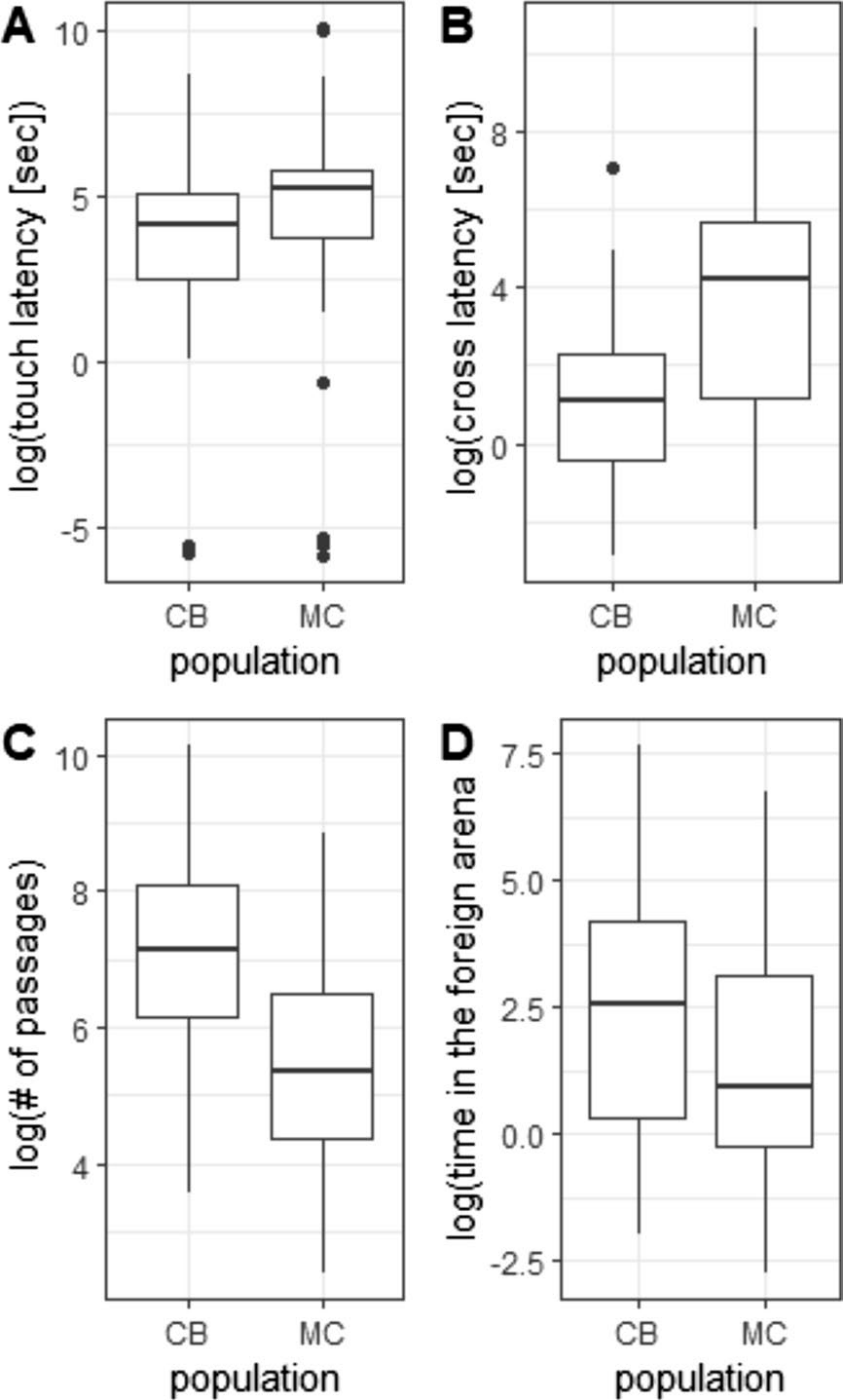
Data are shown log transformed for CB (*n* = 75) and MC (*n* = 70) mice. **A** touch latency, **B** cross latency, **C** Number of passages between the arenas and (**D**) the time spent in the foreign arena. Boxplots show show the medians (horizontal lines), as well as the 25% and 75% quartiles, whiskers represent extreme values and open circles indicate outliers

In more detail, CB mice showed significantly shorter touch (Wilcoxon test: W = 3567, *p*-value < 0.001) and cross latencies (Wilcoxon test: W = 3980, *p*-value < 0.001) than MC mice (Fig. 5, Table 4). Hence, MC mice needed more time to detect the first antenna and also needed more time to enter the other arena. Further, CB mice visit the MC enclosure more often (Wilcoxon test: W = 1125, *p*-value < 0.001) and spent more time in there (Wilcoxon test: W = 2096, *p*-value = 0.037, see Table 4 and Fig. 5 for visualization).

**Table 4:** Number of individuals (males and females) per population and mean, sd, median minimum and maximum values for different weight and four behavioral parameters are given

Population		CB	MC
N total		75	70
N males		41	38
N females		34	32
Weight [g]	Mean ± sd	20.74 ± 4.6	20.06 ± 4.1
	Median	19.48	20.12
	Min/max	14.1/31.8	12.6/30.3
Total # of sessions	Mean ± sd	2743.03 ± 4346.38	611.73 ± 1070.78
	Median	1273	209.5
	Min/max	36/25197	11/6953
Touch latency [sec]	Mean ± sd	200.91 ± 699.05	926.91 ± 3770.22
	Median	65.07	187.3
	Min/max	0/5374.4	0/23360.23
Cross latency [sec]	Mean ± sd	30.68 ± 136.66	1113.71 ± 69.74
	Median	3.04	69.74
	Min/max	0.06/1167.69	0.11/43170.53
Time in foreign arena [h]	Mean ± sd	119.86 ± 323.73	77.15 ± 190.51
	Median	12.64	2.55
	Min/max	0.14/2106.73	0.07/849.14

### Spatial use in the enclosures

Spatial use in the enclosures was monitored as the time spent in the home and foreign arena and the number of passages between the two arenas. We could unequivocally determine the mice being in the one or other arena on average for 89.2% of their total time in the experiment. The missing 10.8% mice spent time either in the tubes, but also technical issues as e.g. one antenna didn’t detect the transponder and thus the mice couldn’t be assigned to one of the arenas, were included here. Generally, mice preferred their home arena over the foreign arena (93.4% home vs. 6.6% foreign). The time mice spent in the foreign arena was significantly correlated with the number of passages between the arenas for all individuals (Pearson correlation: r = 0.21, *p* = 0.011). In general, CB mice showed more passages and more time in the foreign arena (Table 4, Fig. 5) than MC mice.

## Discussion

In this study we investigated the competitive and invasive potential between individuals of two populations of the western house mouse. Both populations colonized Western Europe ~ 3000 years ago [19] following presumably the same colonization route via the Mediterranean Basin [37, 38]. Judged on their pattern of molecular divergence [33–36, 42–44], both populations split most likely soon after the arrival in southern France. We aimed to elucidate whether individual competitive ability varies not only within populations, but also between them. We used two different tests, first, a controlled cage experiment with one-by-one encounters of two males from either population; second, an enclosure experiment, where both sexes and different age groups of the populations were facing each other under more natural conditions and after having established their own territory. The cage experiment revealed a clear competitive bias between the males of the two populations and the same bias was found for males and females during the enclosure experiment. Individuals of the CB population tend to be less restricted in their space use in both tests, and maybe even prevent individuals from the MC population entering their home cage or arena. This behavior has also fitness consequences with respect to higher reproductive success in the mixed mating events, i.e. mating events in which CB males have higher access to the female MC resources than vice versa.

### Competitive ability is rather based on population-specific genetic differences than physical attributes

Many factors, such as food (quantity, quality, distribution), nest sites, space and/or mating partners, are described in the literature to influence or to be correlated with competitive ability in various animal species [17]. Competitive biases between individuals should facilitate either defending or capturing these resources and should thus directly relate to fitness [1, 3, 5, 6] i.e. it should be evolutionarily optimized in any population. Cunningham and colleagues [45] have previously studied competitive behavior in male house mice in semi-natural enclosures and identified competitive ability being heritable and correlated to body mass. In our survey we included individuals of two allopatric populations CB and MC, which have already been extensively described and which exhibit clear genetic differences [33–36, 44], as well as differences in other behavioral components [25, 26, 46]. We observed a strong competitive bias towards males of the CB population. This was not dependent on body size, however we found an effect of age in the controlled cage experiment. As house mice in general arrived in Europe via two different colonization routes, either through central Europe or via the Mediterranean Basin, both focal populations potentially followed two different routes and differences in their competitiveness might result from independent evolution of both populations. However, Bonhomme et al. 2011 investigated the colonization of Europe by house mice from > 30 populations by using the mitochondrial D-loop, including one population from France and one from Germany. Both populations share the same D-loop haplogroups, suggesting a same matrilinear history and thus sharing the same colonization route. A study by [40] investigated several French and German populations (including mice from the same MC and CB region as used in this study) and included them into the dataset by [41]. Haplogroups based on the mitochondrial D-loop of the MC and CB population of [40] fall into those French and German populations found by [41], describing the same matrilinear footprints via the Mediterranean Basin. Thus, it is most likely, that both focal populations in this study followed the same colonization route and colonized Europe ~ 3,000 years ago [19]. Hence, we attribute the difference in the competitive behavior to population-specific genetic differences—despite their very recent divergence time of not more than 3,000 years—and not to physical strength.

### Space use and territoriality

Space is one of many resources, which individuals compete for, as with it, more other resources might be reached and allows populations to expand further. Our results suggest that the more competitive individuals are less restricted in space use than submissive individuals. With our enclosure experiment we aimed to mimic a social community of its own population before they became connected to the other population. We found that, individuals of the CB population spent more time in the foreign territory and visited the foreign arena more often. The long touch and cross latency of MC individuals might result from CB individuals retarding the MC individuals to cross, either through olfactory territory marks [20, 47] at the entrance of the arena and/or by physical presence. On the other hand, there are differences in individual behavior in each population, i.e. also some MC individuals did visit and explore the respective other arena and some CB individuals were far less exploratory, which is consistent with the assumption that the competitive ability has also a broad variation within each population.

At the end of the experiment, we found individuals with pure but also with mixed ancestry, thus individuals of both populations had reproduced and also found partners of the respective other population. More pure than mixed ancestry offspring occurred, indicating some degree of assortativeness [26]. However, most mixed population background offspring had been sired by CB males, suggesting a direct reproductive advantage, at least during the early encounter phase. It was suggested for female house mice to prefer dominant vs. subordinate males [7, 47–49], which supports our finding.

### Competitive ability is a fast-evolving trait in house mice

Our results show a clear behavioral divergence between the two tested populations, raising the question how this competitive disparity may have evolved. Since we have no information on the originally colonizing population, one cannot directly infer whether the higher or the lower competitiveness is the derived trait. Still, when taking the colonization history into account one can speculate that the higher competitiveness is associated with the expansion front. The arrival of mice in Southern France and the further spread into the North might have favored more exploratory individuals to spread faster. Assuming that this behavior has a genetic basis [45], this would lead to more competitive populations in the North. This could have been supported by the effect of allele or gene surfing [50–52], which assumes that populations at the expansion front have a lowered effective population size, where allele frequency changes might occur more easily. Hence, the speed of evolution in these populations is increased and underpins our suggestion that competitive ability can be a fast-evolving trait. Nevertheless, genetic drift might act against selective changes, especially in small populations and the rate of evolution might be increased in bigger populations with higher genetic variation. In evolutionary and population genetic studies, the behavioral factor is often underestimated, as it is mostly attributed only to individuals. Of course, individuals’ behavior can only influence and alter the properties of a whole population, if it is present in many individuals of the respective population, which is the case in our example, but this needs to be accounted for in evolutionary models. A behavioral shift might have different effects on the rate of evolution [14] of these populations, as it might decrease the rate when individuals are able to avoid selection pressures either due to new possibilities how to interact with their environment but also due to the ability to move to a new environment. However, this is assumed to be on a short-term scale. In a more long-lasting view, the evolutionary rate would be increased by being exposed to novel selection pressures either in the old or the new environment. Based on the nucleotide diversity, the rate of evolution between the MC and CB populations seem to be comparable [42], however the estimator used (Watterson's theta (Ɵ_W_)) does not take allele frequencies into account.

Duckworth [14] points out the deficit general population genetic models have in this respect, as these accept behavior to have an influence on the evolution of populations in general, however, as it is seen as mostly an individuals’ property it is excluded by assuming no migration and random mating. Not many studies exist, which link the behavior of individuals to populations. However, one example in house mice, described by Montero and colleagues [26], is the fast evolution of recognition cues involved in mate choice behavior in house mice, where they described a non-random mating pattern between two populations of house mice, apparently determined by paternally provided cues.

## Conclusion

This study provides insights on how individual behavior could impact the evolution of the complete population. In our study we detect that individual competitiveness has a population wide effect in the disparity of competitive and exploratory ability between two house mouse populations of the same subspecies and was able to evolve during a colonization situation. We propose that the rapid spread of the mice after their arrival, could have favored more competitive populations at the expansion front. This shows the impact behavior may have on the evolution of populations and how we can envisage the evolution of populations in a meta-populational context.

## Methods

### Wild mice

We used wild-derived house mice, *M. m. domesticus* from Western Europe. These mice originated from wild-caught individuals trapped in 2005 and 2006, in two locations – the Massif Central region in France (MC) and the area around Cologne/Bonn in Germany (CB). Since then, both populations, starting with 10–15 breeding pairs, were kept under an outbreeding regime, following the HAN- rotation system [53], in the breeding facilities of the Max Planck Institute for Evolutionary Biology in Plön, Germany [42]. At the time of the experiments, mice breeding was in the 5th generation.

Both populations colonized Western Europe ~ 3000 years ago [19, 41] following most likely the same colonization route via the Mediterranean Basin [40, 41]. Since then, both populations had time to diverge. Most likely, both populations split soon after the colonization, based on the pattern of molecular divergence [33–36, 42–44]. The encounter experiments (see below) were conducted by individual monitoring of 15 pairs of mice. The focal mice from encounter experiment of the MC and CB population originate from 6 and 7 families, respectively, in total from 19 different litters. All mice were weaned at an age of 4 weeks and they stayed together in their brother groups until one day before the experiments, when they moved into the test cage. Each encounter consisted of two adult males (aged between 288 and 401 days), one from each population (MC and CB). Even though mice were already a little older, wild mice are still reproductive active at this age. The two setups (in room #113 and room #114) started with 107 adult individuals in total (more information in the detailed description of the enclosure experiment further down).

The experiments were performed 2013–2015. This work did not involve research on animals that would require permission by an ethics committee. The keeping and handling of the animals was done under the permission of the authorities (permit from Veterinäramt Kreis Plön: 1401–144/PLÖ-004,697), according to §11 of the German animal welfare law (Tierschutzgesetz).

### Experimental Design

To investigate competitive ability and its influence on fitness we used two different approaches: 1) a controlled cage experiment, with individual monitoring of behaviors of male dyadic encounters, 2) an enclosure experiment, in which mouse movement was tracked via RFID-transponders, followed by microsatellite-based population assignment.

### Encounter experiment

**Fig. 6:**
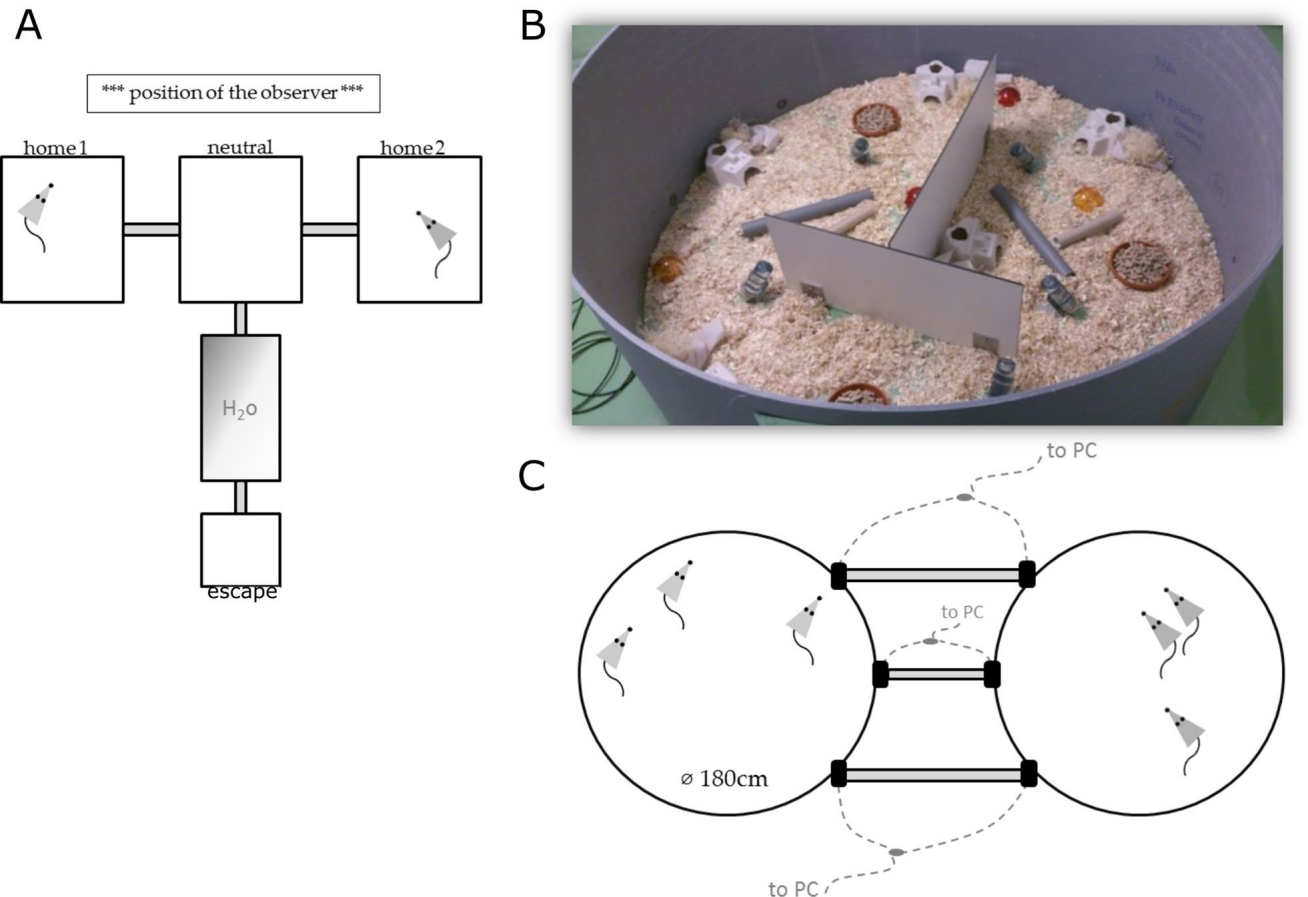
Schemes of experimental setup. **A** Scheme of the controlled cage experiment, **B** photo of one arena of the enclosure experiment, **C** scheme of the enclosure experiment

To investigate the competitive behavior between males of the two populations we ran fifteen one-by-one encounter experiments in a controlled cage setup. The experimental setup consisted of two satellite cages (later referred to as “home cages”, one for each individual) and one neutral cage in the center, to which an escape cage was connected via a water bath (Fig. 6A).

The escape cage gave the individuals the possibility to seek a shelter in case of too much aggressiveness during the encounters themselves. However, since it should not be used as a normal home cage, it could only be reached through a water bath as obstacle. All cages were connected with plexiglas tubes. Each of the cages was equipped with food and water and a small house as shelter. After each test run the complete setup was cleaned and disinfected. Each pair was tested twice for 10 min each on two consecutive days. To avoid possible side effects due to setup orientation in the room, we reversed the placing of the individuals into the home cages on the second day. Experiments were conducted between 9 and 12am. We did not include any habituation phase to the setup, as we did not want the individuals to meet before the experiment. The dyads were arranged such that we could exclude weight as potentially influencing the competitive bias. We did not balance the age of the partners in these experiments, but analyzed it as possible factor.

The behavior of two individuals (one male of MC and one of CB origin) was monitored simultaneously by one observer (N.R.). All behaviors counted were listed and described in detail in Additional file 1, included were agonistic, submissive as well as neutral behaviors. The locations of each of the two focal individuals in the setup (home cage, foreign cage, neutral cage, escape cage or the respective plexiglas tubes) were noted every 15 s.

Agonistic behaviors were of special interest (see Additional file 1 for details) for the question of dominance behavior. Agonistic interactions were either offensive or defensive towards the respective other individual. Further, these conflict situations could be classified into either “decided” or “undecided” conflicts. Decided conflicts were situations where one individual showed defensive behavior towards the other (e.g. one individual being attacked and flees), in undecided situations none of the individuals exhibited defensive behavior (e.g. both individuals stayed).

### Statistical analysis of the encounter experiments

To identify a potential competitive bias between the two dyadic partners, i.e. to determine whether there was a clear “winner” (i.e. dominant individual) or “loser” (i.e. submissive individual) in the dyadic pairs, we used a Binomial Test on the number of decided conflicts between individuals per dyad, as well as the number of dyads in which CB individuals won over MC. To identify a potential population effect, we included population background (MC vs. CB), but also body weight and individual age as possible factors influencing the outcome of the single encounter experiments. The number of conflicts was summed up over both experimental days and was used as response variable for a statistical model. As our data is count data, we first chose a poisson distribution. However, a “zero inflated negative binomial poisson regression model” showed the best fit to our data, checked for by using the Shapiro–Wilk test to test for normal distribution of the model residuals (W = 0.9477, *p* = 0.205) and by visual inspection of QQ-plots. We chose a negative binomial distribution as alternative to the regular poisson distribution as this is recommended for over-dispersed data, i.e. where the variability in the data is greater than expected with a given distribution.

Further, we ran a principal component analysis based on all behaviors which contributed to > 5% of all behaviors for all pairings. As the dataset is count data and not normally distributed, we calculated Bray–Curtis distance between individuals and ran an ANOSIM analysis with 999 permutations and population background as grouping factor, to identify a potential effect of population background on competitive predominance. In order to see if weight or age of individuals affect competitive ability we used Mantel Tests based on the Euclidian distance between individuals, with 9999 permutations.

Further we analyzed the use of the three main cages (foreign, home and neutral cage) for dominant and submissive individuals and between MC and CB with a Friedman Rank Sum test, as this non-parametric test is applicable to paired data with three or more possibilities. Pairwise comparisons between the different cages of each set of individuals (dominant vs. submissive or MC vs. CB) Comparisons between MC versus CB individuals were performed using the Wilcoxon Test, a non-parametric test for paired data with two samples. Multiple testing was addressed by Bonferroni correction.

For visualisation and statistical analysis we used the program R [54] and its packages “ggplot2” [55], “vegan” [56] and “pscl” [57].

### Semi-natural enclosure experiment

Complementary to the controlled cage experiment we conducted an enclosure experiment in duplicate rooms (room #113 and room #114). Each setup consisted of two round arenas (diameter 180 cm, 70 cm apart, see Figs. 6B and 1C), which were connected via three plexiglas tubes. Each of these tubes was equipped with two RFID-antennae, one at each entrance to the respective arena. Each arena was settled by individuals of the French (MC) or German (CB) house mouse populations described above. The connections between the arenas were initially closed. All mice used for this experiment were born and raised in their respective home arena before opening the connections, allowing them to become fully acquainted in the arena. Adult founder animals were ear clipped and tagged with a passive RFID-transponder (Iso FDX-B, Planet ID).

It was important that experimental mice were already born in the setup, as we had previously found that the initial behavior in the arenas is different if mice are introduced from cages (coming from the breeding facility) from the long-term behavior [26]. Therefore we introduced MC and CB “cage—born—individuals” to enclosures (as described in [26] and let them breed without interference (starting August 2013, see Table 5).

**Table 5:** Time line of breeding time and the semi natural enclosure experiment

Date	Experimental phase
August 2013	Start breeding mice for enclosure experiment (MC and CB separated in two different rooms)
July 2014	7–8 males and females were chosen from the breeding rooms and placed into the MC and CB arena. These mice represent the parents of our later experimental mice
July–December 2014	Health checks, counting of individuals and transponder the animals which are finally to be used in the experiment; remove all other adult individuals
December 2014	START of the experiment; open connections between the arenas in both rooms
January 2015	Health check, counting of individuals, DNA sampling and transponder offspring
March 2015	END of the experiment

In July 2014, we randomly picked 7–8 individuals per sex and population and distributed them into the respective enclosures of both rooms, still divided by MC and CB population. In their respective arenas, again, individuals reproduced and we only disturbed them for health checks, counting of individuals and placing the RFID transponders. In December 2014 a sufficiently high number of experimental mice was reached, and all older individuals which were in the enclosures for breeding the experimental mice were removed; the experiment started by opening the connections between the arenas.

After four weeks, the connections between the arenas were closed for a few hours and all animals were caught in order to check the health status, to tag further grown up offspring individuals and to sample tissue for DNA extraction and later population assignment. The experiment was run for a total of 12 weeks, until mid of March 2015. During this time mice had the chance to freely mate and reproduce.

### Microsatellite genotyping and population assignment

All individuals from the enclosure experiments were genotyped using 13 unlinked microsatellite markers. Markers were taken from [26]. Forward primers were labeled with FAM or HEX and PCR was performed using 5 ng/µL DNA template together with the Multiplex PCR kit (QIAGEN). After processing PCR products with HiDi formamide and 500 ROX size ROX standard, samples were run on an ABI 3730 sequencer (Applied Biosystems). Raw alleles were called using GeneMapper 4.0 (Applied Biosystems). Microsatellite information can be found in Additional file 2. Microsatellite information is missing for 2 individuals of room #113 and for 9 individuals of room #114.

Based on the microsatellite genotypes, STRUCTURE analysis (STRUCTURE 2.3.4 [37, 38]) was performed to determine the population of origin of all offspring in the enclosures and was used to determine whether offspring was of pure (either MC or CB) or mixed population origin (one parent of each population). Further, we used CERVUS [39] for parentage analysis for mixed-background offspring. However, parentage could not be assigned unequivocally. Thus, to infer maternal/paternal population background of mixed origin individuals, we investigated the population background of the two most likely parents and accepted maternal/paternal population assignment if both most likely parents showed the same population origin. In room #113 and #114 there were 18 out of 34 and 26 out of 53 female individuals among the two likely mothers and 11 out of 35 and 19 out of 38 males among the two most likely fathers.

### Data processing and statistical analysis for the enclosure experiment

All founder mice (*n* = 107; see Table 2: sum of all adult individuals at the beginning of the experiment) and new mice found during the health check after four weeks (*n* = 54) received a passive RFID transponder such that information about migration between the arenas was gained for each mouse. The mice found during the health check were the “young” individuals found at the starting phase (*n* = 67, see Table 2: sum of all young individuals at the starting phase), however not all survived until the health check. Analyses were performed on in total 145 individuals (70 MC and 75 CB individuals), including founder (*n* = 102) and first offspring (*n* = 43) individuals. 16 individuals (5 CB individuals, 11 MC individuals) were never registered at any antenna and thus were excludedas the RFID Transponder were either not read and mice were eventually found dead.

Every time a mouse passed one of the antennae, it was registered; a time stamp and the identification number of the respective antenna were saved into a text-file. After processing the raw data files, the following parameters could be assessed for each individual: number of passages between the two arenas, the time mice needed to detect the entrance to the other arena, i.e. were registered for the first time (touch latency), the duration between the registration at the first antenna to the second antenna at the respective other arena (cross latency), and the total time spent in the home and foreign arena. For statistical analysis we used a PERMANOVA (PERmutational Multivariate ANalyses Of Variance; Adonis function of the R package vegan [56]. To analyse competitive behavior and its influencing factors, we included touch and cross latencies, as well as the number of sessions and the time in the foreign arena as dependent variables and weight, sex and population background as explanatory variables. Additionally, we performed Wilcoxon tests to specifically test for differences for each of the four measured parameters between the CB and MC population [56].We did not differentiate between founder mice and mice, which were detected first during the health check as those were most probably sired in the phase before the actual experimental start, thus before opening of the connections. Data processing and statistical analysis was done using self-written R scripts [54], packages used are “vegan” [56] and “ggplot2” [55] for visualization.

## Supplementary Information

**Additional file 1. **Description of all observed bahviors for the dyad- experiment

**Additional file 2. **Microsatellite information for all individuals from the enclosure experiment

## Data Availability

The data used and analyzed during the study are included in this article and its supplementary information files. Raw data have been deposited at Dryad: 10.5061/dryad.ksn02v782.
